# Heavy rainfall increases nestling mortality of an arctic top predator: experimental evidence and long-term trend in peregrine falcons

**DOI:** 10.1007/s00442-013-2800-y

**Published:** 2013-10-18

**Authors:** Alexandre Anctil, Alastair Franke, Joël Bêty

**Affiliations:** 1 Université du Québec à Rimouski et Centre d’études nordiques, 300 Allée des Ursulines, Rimouski, QC G5L 3A1 Canada; 2 Canadian Circumpolar Institute, University of Alberta, Edmonton, AB T6G 2H8 Canada

**Keywords:** Climate change, Precipitation, Breeding success, Avian predator, Survival

## Abstract

Although animal population dynamics have often been correlated with fluctuations in precipitation, causal relationships have rarely been demonstrated in wild birds. We combined nest observations with a field experiment to investigate the direct effect of rainfall on survival of peregrine falcon (*Falco peregrinus*) nestlings in the Canadian Arctic. We then used historical data to evaluate if recent changes in the precipitation regime could explain the long-term decline of falcon annual productivity. Rainfall directly caused more than one-third of the recorded nestling mortalities. Juveniles were especially affected by heavy rainstorms (≥8 mm/day). Nestlings sheltered from rainfall by a nest box had significantly higher survival rates. We found that the increase in the frequency of heavy rain over the last three decades is likely an important factor explaining the recent decline in falcon nestling survival rates, and hence the decrease in annual breeding productivity of the population. Our study is among the first experimental demonstrations of the direct link between rainfall and survival in wild birds, and clearly indicates that top arctic predators can be significantly impacted by changes in precipitation regime.

## Introduction

Variation in annual breeding productivity has been shown to have considerable repercussions on animal population dynamics (Johnson and Geupel 1996; Gaillard et al. 1998). Understanding the mechanisms that influence annual reproductive success is challenging because numerous factors can interact. Trophic interactions (e.g., predation, cannibalism, competition and food availability) and anthropogenic activities (e.g., disturbance and contaminants) have been identified as potential causes of variation in breeding success in various species (Potapov 1997; Ims and Fuglei 2005; Morrissette et al. 2010). The effect of weather is also regarded as one of the key factors influencing breeding output (Steenhof et al. 1997; Moss et al. 2001), and climatic oscillations have often been correlated with population dynamics of both consumers and prey (Grindal et al. 1992; Forchhammer et al. 1998). Weather can affect individuals, and hence populations, directly (e.g., increasing thermoregulation and movement costs; Machmer and Ydenberg 1990) or indirectly through interactions with biotic and abiotic components of the ecosystem (Grant et al. 2000).

In the context of rapid climate change, weather patterns are predicted to be strongly modified (IPCC 2007). In order to assess the vulnerability of animal populations to such changes, an understanding of the nature of the mechanisms linking weather and individual breeding success is crucial. However, our knowledge of the main climatic factors affecting populations remains limited because the exact causes by which weather affects individuals have been mostly inferred rather than experimentally tested (Redpath et al. 2002; Molnár et al. 2010).

The Arctic will experience, during all four seasons, some of the most severe effects of climate change on the planet (Screen and Simmonds 2010), which will undoubtedly affect animals inhabiting this area (Hunter et al. 2010; Ims et al. 2011). However, our knowledge of the consequences of climate change on arctic wildlife is relatively poor (Post et al. 2009) although some progress has been made in a few well-studied species [e.g., geese (Dickey et al. 2008); seabirds (Gaston et al. 2005); polar bears, *Ursus maritimus* (Stirling and Derocher 2012); lemmings (Kausrud et al. 2008)]. Moreover, some bird groups, such as raptorial species, have received much less attention than others (Møller et al. 2010).

Rain is an important component of weather that is often related to breeding success of avian species (Kostrzewa and Kostrzewa 1990; Skinner et al. 1998). Rainfall at different time periods has been associated with either increases or reductions in nest success rates (Olsen and Olsen 1989; Rodriguez and Bustamante 2003), and also with timing of breeding and duration of the breeding season (Carrillo and Gonzalez-Davila 2010). In addition, negative correlations between rainfall and nestling survival (Potapov 1997; Jovani and Tella 2004; Bionda and Brambilla 2012) have been reported. However, previous studies on this topic were correlative and, to our knowledge, experimental manipulations to clearly test for the direct effect of rainfall on nestling have not been conducted.

The main goal of this study was to investigate the direct effect of rain on nestling survival of a top avian predator of the arctic tundra, the peregrine falcon (*Falco peregrinus tundrius*), nesting in the Rankin Inlet area of the Canadian Arctic. In this population, no temporal trend in apparent survival of adults was observed over the last three decades (Franke et al. 2011), but a long-term decrease in annual productivity, despite concomitant declines in persistent organochlorine residues, has been reported [reduction of 0.65 young per territorial pair between 1982 and 1989 and 2002–2009 (Franke et al. 2010)]. Mortality of nestlings in this population is known to vary with the annual amount of rain recorded during rainstorms (Bradley et al. 1997), and a change in precipitation regime was thought to be the most likely mechanism explaining the recent decline in Arctic-nesting falcon productivity (Franke et al. 2010).

To investigate the mechanisms linking rain and falcon productivity, we used direct observations (i.e., camera monitoring) to determine the main causes of nestling mortality and we experimentally manipulated nest site (using artificial shelters) to test for the direct effects of rain on survival of nestlings. We hypothesized that nestlings that received improved shelter from rain would experience higher survival rates than those that remained unprotected. Finally, we examined historical weather data for evidence of changes in precipitation regime that could explain the observed long-term decline in annual productivity of the breeding population. This study uniquely combined direct near real-time observations, field experimentation, long-term population monitoring data and historical weather data to better understand the vulnerability of an arctic top predator to the effects of ongoing climate change.

## Materials and methods

### Study area

**Fig. 1:**
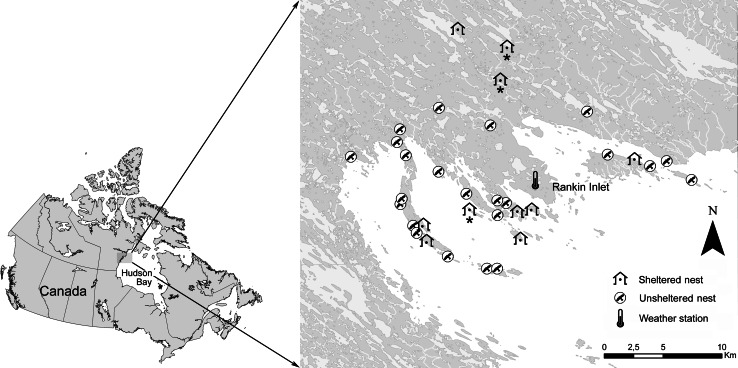
Location of the study site in the Rankin Inlet area, Nunavut, Canada. Detailed study area is shown on the *right panel*, including freshwater bodies (*light gray*), continent (*dark gray*), Hudson Bay (*white*), location of the weather station and positions of sheltered and unsheltered nests of peregrine falcon followed during the nestling rearing period between 2008 and 2010. All sheltered nests were tracked in only 1 year except for three cases (marked with an *asterisk*) that were monitored for 2 years

The study area is located on the west shore of Hudson Bay, near the community of Rankin Inlet (62°49′N, 92°05′W), Nunavut, Canada. Geographically, the region is typified by rolling uplands and lowlands with frequent outcrops of exposed bedrock and eskers. Freshwater lakes, ponds, and wetlands are numerous. The Hudson Bay comprises about half of the study area, and encompasses a barrier island system extending from the mainland as well as several isolated islands (Fig. 1). The peregrine falcon is a cliff-nesting species and suitable nesting sites are found through the study area on islands, along the coast and on the mainland. For more details on vegetation and geology, see Court et al. (1989).

### Study species

The peregrine falcon is a long-lived raptor with world-wide distribution (White et al. 2008). The subspecies *F. peregrinus tundrius* breeding in the Rankin Inlet area is a long-distance migrant and a generalist top predator (White et al. 2008). At peak density, the population reached 29 territorial pairs (one pair per 12 km^2^), making it one of the densest breeding populations recorded for the species worldwide (Franke et al. 2010). Egg laying takes place from early to late June, incubation lasts 33.5 days on average (Burnham 1983) and nestlings hatch asynchronously in July [mean hatch date typically occurs in the second week of July (Court et al. 1988)]. Nestlings are able to thermoregulate independently at approximately 21 days of age (Hovis et al. 1985) and are therefore more vulnerable to cold and wet weather in the first 3 weeks after hatching.

### Data collection

Beginning in May, as falcons arrived from their wintering grounds, territories were surveyed by snowmobile to determine the presence or absence of territorial, breeding-aged adults. All known sites (i.e., cliff or rock outcrop used by a single pair of peregrines including known alternate nest sites within a breeding territory) were checked until occupied or until the breeding season was sufficiently advanced to conclude that the site was vacant (Franke et al. 2010).

#### Camera monitoring

Motion sensitive cameras (RECONYX models PM35T25, PC85 and PC800 Hyperfire; 2009, *n* = 15; 2010, *n* = 17) were installed at a distance of 1–4 m from the nests to determine hatch date and hatch sequence of the nestlings (i.e., hatching order among siblings) during the 2009 and 2010 summers. The nestling’s development stage recorded during the early brood rearing period was used to determine hatch date and hatch sequence in 2008 and for nests with no camera in 2009 and 2010 (see Cade et al. 1996). When triggered, cameras were programmed to immediately capture from one to three photographs followed by a quiet period of 5–15 s (the time period after a trigger during which the camera did not respond to motion events). In addition, cameras were programmed to collect a single time-lapse image every 15 min. Each year cameras were immediately removed from failed nests and transferred to an active nest to ensure that we monitored as many broods as possible. Over both years, nestlings ≤25 days old were monitored with cameras at 23 different nest sites (2009, *n* = 14; 2010, *n* = 15) for a combined total of 588 days (average = 22, minimum = 2, maximum = 28). We analyzed images from the cameras to examine feeding rate of juveniles, exposure to rainfall or other external events, to determine the causes of nestling mortality.

Based on recorded observations, we summarized the causes of mortality into one of four categories; exposure to rain, starvation, other, and unknown. Using the photographs, we analyzed daily feeding events for every nestling 0–25 days of age. We determined that healthy nestlings of <25 days old were typically fed at least three times per day. Therefore, those that were routinely fed on three or more occasions per day, but were exposed to rain (i.e., visibly wet in camera images) and died during the rainstorm were assumed to have died as a result of direct exposure to rain. Conversely, nestlings that were fed fewer than three times per day for 3 or more consecutive days and that did not die during or soon after a rainstorm were assumed to have died from starvation. Nestlings that died of known causes that were not related to rain or starvation were classified as “other” (see “Results”), while nestlings that died for reasons that remained unclear were classified as “unknown.”

#### Experimental nest box manipulation

**Fig. 2:**
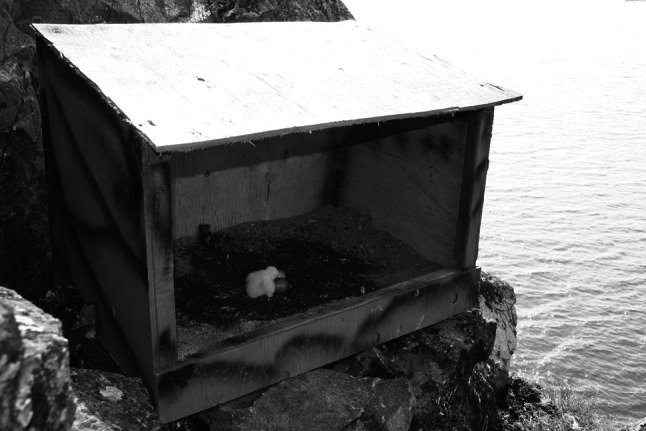
Wooden nest box (56 × 56 × 81 cm) with an opening of 38 × 76 cm used to shelter peregrine falcon nestlings and test for the direct effect of rain on early survival

We deployed 13 (2008, *n* = 2; 2009, *n* = 5; 2010, *n* = 6) wooden nest boxes at nest sites soon after nestlings hatched in order to shelter them from the combined effects of rain and wind during heavy rainfall events. Nest boxes of 56 × 56 × 81 cm with an opening of 38 × 76 cm (see Fig. 2) were painted to resemble the nesting cliffs. Typical nesting substrate consisting of a mix of sand and gravel was added to each nest box to ensure good drainage and to provide sufficient weight to prevent shifting. Sites that received a nest box were selected randomly from those available each year. However, not all occupied nest sites were suitable for a nest box as some ledges were too small for them (2008, *n* = 4; 2009, *n* = 4; 2010, *n* = 3). We nonetheless included these control sites in our analyses because their exclusion generated similar results.

Nest box deployment was attempted only on days when the weather conditions were mild. A shallow depression mimicking the natural scrape that is usually built and maintained by adult falcons was excavated in the substrate and the nestlings were placed into it. A small prey item was placed conspicuously at the entrance of the nest box to encourage adult falcons to return quickly to nestlings and engage in normal feeding behavior. A site that received a nest box was monitored continuously until at least one of the adults had accepted it (i.e., feeding or brooding behavior was observed). Adults usually adopted a nest box very quickly (usually in less than 10 min). However, when pairs (three of 16) failed to return to normal brooding behavior within 1.5 h, the nest box was removed. A prey item was left on the natural nest ledge when deployment of a nest box was abandoned, and the site was monitored to ensure that adults resumed normal nestling rearing behavior. No nests were abandoned following the disturbance. In August 2010, ambient temperature was recorded every 30 min inside and outside six nest boxes using temperature data loggers (Thermochron; accuracy ±1 °C) to verify that sheltered nestlings would be exposed to similar thermal environments as unsheltered ones. Five nest boxes had a positive but weak effect on ambient temperature while one had a weak negative effect (mean difference inside vs. outside nest boxes was 0.3 °C; *t*-value = 15.3, *p* < 0.01, 95 % confidence interval = 0.27–0.35 °C).

#### Nest and weather monitoring

We typically visited nest sites every 5–7 days, on days when weather conditions were mild. Each nestling was color marked using non-toxic, permanent markers to identify individuals. At approximately 25 days, nestlings were marked permanently using unique alphanumeric color-coded and US Fish and Wildlife Service federal bands.

Breeding productivity was calculated as the number of surviving nestlings per territorial pair. A nestling that lived 25 days was considered to have survived. We selected 25 days of age rather than fledging (average 35–40 days) because after 25 days nestlings often move several meters from the nest ledge restricting our ability to document mortality events and causes. The sex of nestlings that died at an early stage was not determined and therefore the potential effect of sex on survival was not taken into account (see “Discussion”).

Weather data recorded at the Rankin Inlet airport were downloaded from the Environment Canada (2011) website. Daily rainfall was retrieved from this meteorological station, which is situated approximately in the middle of the study area (Fig. 1).

### Data analysis

#### Nest box experiment

We modeled the probability of nestling survival using a generalized linear mixed effects model [logit link function in the package lme4 version 0.999375-39 (Bates et al. 2011)] in R Statistical Environment version 2.13.0 (R Development Core Team 2010). We compared survival of nestlings at sites with nest boxes to those on naturally exposed ledges (control nests with no nest box) by modeling the probability of nestling survival (binomial response, lived = 1, died = 0). Models included the variable Nest Box (1 = yes, 0 = no) as well as the covariates Hatch Date (hatch dates transformed as deviation from the median hatch date in each breeding season) and within-brood Hatch Sequence (1st hatch to 4th hatch). To account for variation in survival over time and space, Year and Site were modeled as random effects. In addition, we tested for interactions between Nest Box and Hatch Date, and between Nest Box and within-brood Hatch Sequence. Nine different combinations of these variables were tested and models were ranked based on second-order Akaike’s information criterion (AICc) to control for small sample size. Models with ∆AICc <2 were selected (Burnham and Anderson 2001). We used model averaging to estimate parameters from the selected models to reduce bias and increase precision (Burnham and Anderson 2002).

#### Precipitation

In the same study area, Bradley et al. (1997) reported that mean falcon nestling mortality was correlated with annual precipitation recorded during storms (defined as ≥3 days of consecutive rain). However, our camera data indicated that weather-related mortalities occurred during very short periods (<24 h, but sometimes within as little as 2 h when not brooded) of intense rainfall. These observations allowed us to identify days during which some nestlings died because of direct effects of rainfall. Local weather data recorded on those days indicated that 8 mm was the daily minimum amount of rain that caused mortality. We thus used this value as the “precipitation threshold” (daily minimum amount of rain) that was known to be associated with nestling mortality during our study period (hereafter referred to as “heavy rain”). As lower amounts of rain could possibly cause nestling mortality, we performed sensitivity analyses using lower threshold values to evaluate the strength of our conclusions. We calculated the number of days of heavy rain in July and August for all years of the study (i.e., 1981–2010). We used July and August because this period encompasses the 25-day time frame when nestlings are considered to be most vulnerable to weather (Hovis et al. 1985). We used linear regression to examine temporal trend in the annual number of days with heavy rain from 1981 to 2010.

We examined the relationship between the number of days with heavy rain and nestling survival using a generalized linear model. Actual number of surviving nestlings was used as the dependent variable while the total number of nestlings at hatch was used as an offset. We limited our analysis to 17 years (1982–1995 and 2008–2010) as the number of nestlings at hatch was not recorded in other years. We used a similar model to test for a temporal trend in nestling survival before and after controlling statistically for the effect of heavy rain. We also used a generalized linear model to test for temporal trends in the number of eggs laid with territorial pair as an offset, the number of nestlings at hatch with the number of eggs laid as an offset and annual breeding productivity (number of young produced) with territorial pair as an offset. Finally, we also tested for a temporal trend in annual breeding productivity after controlling statistically for the effect of heavy rain. For all analyses, data were log transformed to achieve normality (Kolmogorov–Smirnov, all *p* ≥ 0.2) and graphical inspection of the residuals revealed no trend.

For all analyses testing for temporal trends, we computed the Durbin–Watson statistic to test for autocorrelation problems, which could bias the parameter estimates of the regression (Box et al. 1994). However, no autocorrelation was detected for any of the tests. All analyses were done in the R Statistical Environment.

## Results

### Causes of mortality

Motion-sensitive cameras captured the death of 26 nestlings (<25 days old; 2009, *n* = 11; 2010, *n* = 15) from 14 broods (2009, *n* = 5; 2010, *n* = 9). In 2009, the resident female of a nest site vanished well into the nestling rearing period, which is a highly unusual event in our study population. Her three nestlings died a few days later after not being taken care of. The female was likely killed and although we included her nestlings’ deaths in the mortality summary, we decided to exclude this nest from all other analyses.

Overall, 38 % (10/26) of deaths were caused by the direct effect of rainfall. These mortalities occurred in 16 % of the nests (5/31) and affected one to four nestlings per nest. This includes a nestling that was knocked off of the cliff by the resident female as she flew from the nest ledge while brooding three large nestlings during a heavy rainstorm. Four nestlings (15 %) died as a result of starvation and four other (15 %) from events not related to either rain or starvation. These included the three nestlings of the female that was likely killed (see above) as well as a nestling that died shortly after being bitten and grabbed aggressively by the resident female. Finally, eight deaths (31 %) occurred due to reasons that could not be confirmed (though a combination of rainfall and starvation were suspected in four cases). Four deaths were recorded for nestlings raised in a nest box, but none were attributed to rainfall. Of these, one was killed by the female and three died of an unknown cause clearly not related to the direct effect of rain. Moreover, there were no nestling deaths attributed to predation in either sheltered or unsheltered nests.

### Nest box experiment

**Fig. 3:**
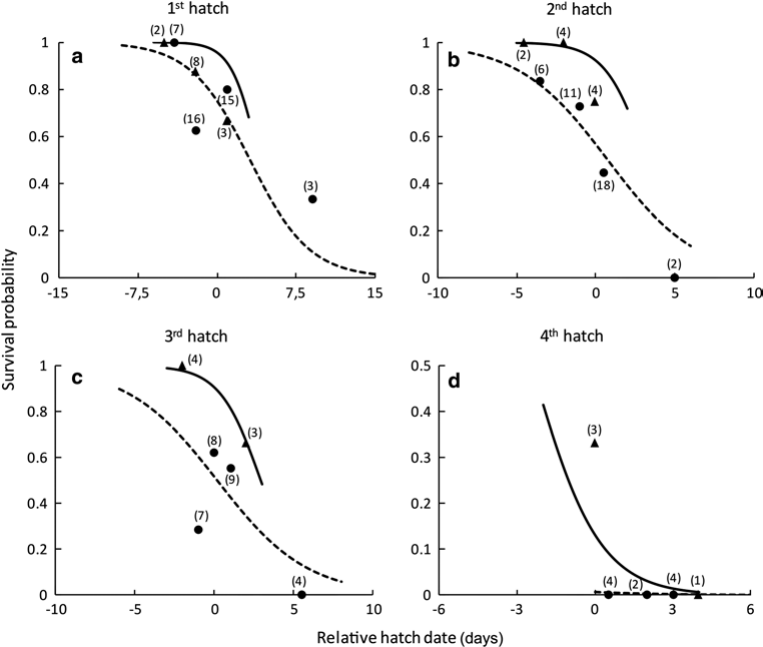
Survival probability of Arctic-nesting peregrine falcon nestlings up to 25 days old in relation to their relative hatch date (values are standardized relative to the yearly median) and within-brood hatch sequence (**a**–**d**). *Lines* represent values of the fitted logistic regression model [nestlings sheltered in a nest box (*solid line*), unsheltered nestlings (*dashed line*)]. Values were obtained using the average random effect calculated for each hatching position, with or without shelter, separately. To illustrate the adequacy of the model, *each point* represents the proportion of surviving nestlings grouped by similar hatch date [sample size is shown near each point; sheltered nestlings (*triangles*), unsheltered nestlings (*circles*)]

Over 3 years (2008–2010), we followed 34 nestlings from 13 broods raised in nest boxes and 117 nestlings from 41 broods raised on natural ledges. We found strong evidence for an effect of hatch date on nestling survival (Table 1; Fig. 3). Nestlings that hatched later in the season had much lower survival than those that hatched earlier. For example, the survival probability of the first-hatched nestlings not raised in a nest box was on average 97 % lower in late-hatched chicks relative to those hatched 24 days earlier (Fig. 3). Within a given brood, nestlings that hatched first, second or third had similar survival probability. However, young that hatched fourth experienced lower survival than first-hatched nestlings (Table 1; Fig. 3).

**Table 1:** Variables, number of parameters (*k*), second-order Akaike’s information criterion (*AICc*), ΔAICc, AICc weight (*AICcWt*) and log-likelihood (*LL*) of the candidate models explaining peregrine falcon nestling survival, and model-averaged parameter estimates from the two most parsimonious models, unconditional SE and 95 % confidence intervals (*CI*; lower CI and upper CI)

Selected models					
Variables	*k*	AICc	ΔAICc	AICcWt	LL
Nest box, Hatch date, Hatch sequence	8	160.93	0.00	0.51	−71.96
Nest box, Hatch date, Hatch sequence, Hatch date × Nest box	9	161.97	1.04	0.30	−71.35
Nest box, Hatch date, Hatch sequence, Hatch date × Nest box, Hatch sequence × Nest box	12	164.28	3.35	0.10	−69.01
Nest box, Hatch date, Hatch sequence, Hatch sequence × Nest box	11	165.68	4.75	0.05	−70.89
Nest box, Hatch sequence	7	166.51	5.58	0.03	−75.86
Hatch date, Hatch sequence	7	169.27	8.34	0.01	−77.24
Hatch sequence	6	176.16	15.23	0.00	−81.79
Hatch date, Nest box	5	176.81	15.88	0.00	−83.20
Hatch date	4	178.85	17.82	0.00	−85.24

Parameters	Nest box	Hatch date	Second hatch	Third hatch	Fourth hatch	Hatch date × Nest box	Intercept
*Β*	2.45	−0.35	−0.84	−1.08	−5.77	−0.42	0.96
SE	0.98	0.13	0.56	0.63	1.73	0.42	0.54
Lower CI	0.54	−0.61	−1.94	−2.31	−9.15	−1.25	−0.10
Upper CI	4.36	−0.10	0.26	0.14	−2.38	0.41	2.02

A random effect of Year and Site is included in all models

*Nest box* presence or absence of a nest box, *Hatch date* value relative to the annual mean hatch date, *Hatch sequence* within-brood hatch order (1st, 2nd, 3rd and 4th; 1st hatch is included in the intercept), *Hatch date × Nest box* interaction between the hatch date and the presence of a nest box

After controlling for the effect of hatch date and within-brood hatch sequence, nest boxes had a positive influence on survival (Table 1; Fig. 3). We also found some evidence (second-best model) that the positive effect of a nest box was reduced in nestlings that hatched relatively late in the breeding season (Table 1).

### Precipitation

**Fig. 4:**
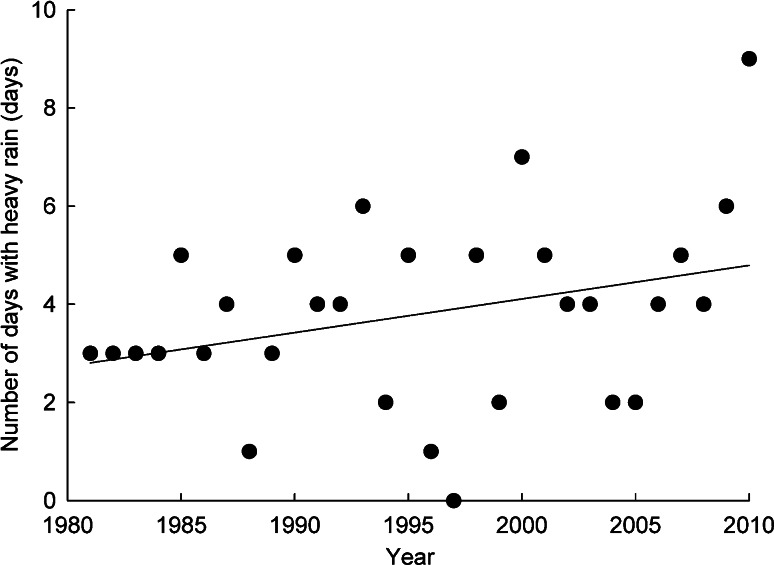
Number of days with heavy rain (≥8 mm/day) recorded by the Rankin Inlet airport weather station in July and August between 1981 and 2010

Using scouting cameras, we detected mortality as a result of the direct effects of rain (see above) on four distinct rain events (2009, *n* = 2; 2010, *n* = 2). The frequency of heavy rain events has shown an increasing trend from 1981 to 2010 (*β* = 0.07; similar trends were found with thresholds of 5–7 mm of rain, *β* = 0.08–0.12). Overall, about 2 additional days of heavy rain were recorded in recent years compared to the early 1980s (Fig. 4). For the period 1981–2010, the annual rainfall recorded for July and August showed an increasing trend of about 3.1 mm per decade. The mean daily temperature recorded in July and August was 10.1 °C and also showed an increasing trend (*β* = 0.055 °C/year).

**Fig. 5:**
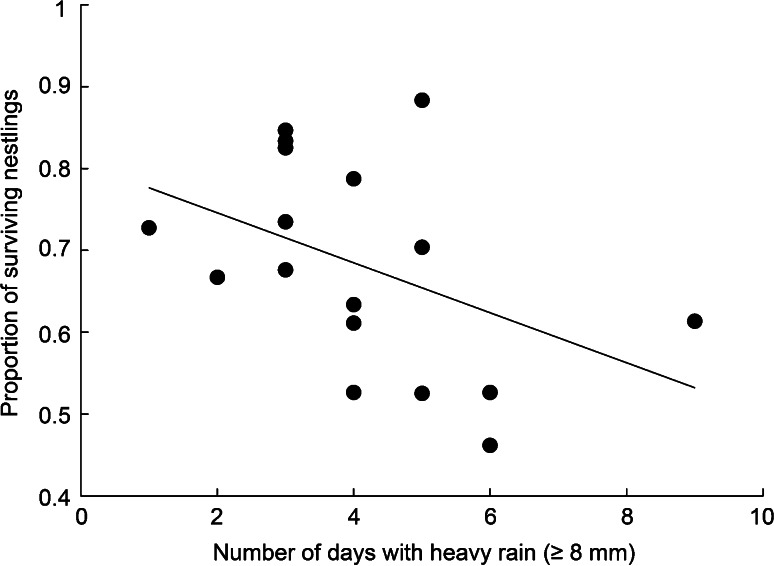
Relationship between the number of days with heavy rain (≥8 mm/day) recorded in July and August, and the proportion of peregrine falcon nestlings surviving up to 25 days old in the Rankin Inlet area (1982–1995 and 2008–2010). To illustrate the adequacy of the regression model (*line*), we show the annual proportion of surviving nestlings (*points*). The annual number of nestlings at hatch varied from 26 to 77 (mean 44)

Nestling survival during the study period tended to be lower in years with more days with heavy rain (*β* = −0.03, SE = 0.02, *p* = 0.08, *R* ^2^ = 0.19, *n* = 17; Fig. 5; similar trends and slightly better fit were found with critical daily thresholds of 5–7 mm of rain, *β* = −0.02 to −0.03, SE = 0.01, *p* = 0.02–0.05, *R* ^2^ = 0.23–0.32). No significant relationship was found when we used the total annual amount of precipitation recorded during the nestling rearing period (*β* = −0.001, SE = 0.0008, *p* = 0.19, *R* ^2^ = 0.11).

We found a long-term temporal decrease in nestling survival (*β* = −0.013, SE = 0.005, *p* = 0.01, *n* = 17 years). However, this trend was no longer statistically significant (*β* = −0.011, SE = 0.006, *p* = 0.10) after controlling for the effect of frequency of heavy rain (i.e., by using the variable year and number of days with heavy rain in the same model). This suggests that the temporal decline in nestling survival partly resulted from the increase in the frequency of heavy rain in recent years. Using the same 17 years of data, we found no temporal trend in the number of eggs laid per territorial pair (*β* = −0.004, SE = 0.004, *p* = 0.34) or in the number of nestling at hatch per egg laid (*β* = −0.01, SE = 0.007, *p* = 0.12), but a temporal decrease in annual breeding productivity was found (*β* = −0.03, SE = 0.01, *p* = 0.01). However, this negative trend was no longer significant (*β* = −0.02, SE = 0.01, *p* = 0.19) after controlling statistically for the effect of heavy rain.

## Discussion

Although the greatest impacts of climate change are expected to occur in the Arctic (Screen and Simmonds 2010), little is known about the mechanisms linking weather to reproduction and survival of most Arctic-breeding species (but see Dickey et al. 2008; Kausrud et al. 2008; Stien et al. 2012; Stirling and Derocher 2012). This is especially true for arctic predators, which are seldom studied. The direct effects of weather have long been suspected to influence early survival in northern wildlife (e.g., Stirling and Smith 2004; Mallory et al. 2009). In peregrine falcons, rainfall has previously been identified as a potential driver of nestling mortality (Ratcliffe 1984; Mearns and Newton 1988; Olsen and Olsen 1989). Using a short-term experiment combined with camera-recorded observations and a long-term database, we found that the direct effect of rain accounted for a high proportion of falcon nestling mortalities in an Arctic-breeding population. Despite a relatively small sample size, our field experiment showed that protecting nestlings from direct exposure to rainfall significantly increased their survival. In addition, we found strong evidence for the link between the increase in the frequency of heavy rain and the long-term decline in annual reproductive success of the study falcon population. In this regard, our detailed study provides crucial information in the assessment of the vulnerability of a top arctic predator to climate change.

The lower survival rate of nestlings exposed to heavy rain compared to those that were sheltered is, to our knowledge, the first experimental demonstration of the causal mechanism linking precipitation and nestling survival. Although nest boxes likely sheltered nestlings from a combined effect of rain and wind, no deaths were attributed to wind alone, while some deaths attributed to rain occurred on days with relatively slow wind speed. This suggests that, although wind likely increases the effect of rain, protection from rainfall is the main reason explaining increased survival in sheltered nestlings. During heavy rain events, cameras recorded unsheltered nestlings often getting wet unless very well protected by the parent. Indeed, parents can brood their young for several hours, but camera data showed that even short absences can lead to nestling mortality. Wet downy feathers lose their insulation properties and therefore heat loss increases rapidly (Nye 1964). On the other hand, sheltered nestlings remained dry most of the time even in the absence of an adult. In such cases, siblings would huddle together to conserve heat.

Although the sex of an individual can influence survival in dimorphic species (Roskaft and Slagsvold 1985), we did not include this variable in our analyses as individuals that died at an early age could not be sexed using external cues. However, we are confident that it did not generate a bias in our study as we deployed the nest boxes randomly (and hence the sex ratio after hatch was likely similar between treatments). Moreover, sex-biased mortality is often due to a difference in food requirement (Roskaft and Slagsvold 1985) and our study was limited to the first 25 days after hatch, a period during which males and females have similar feeding rates (Boulet et al. 2001).

The effect of hatch date on nestling survival was much stronger than that reported in most bird studies (e.g., Dawson and Clark 1996; Riley et al. 1998), but was similar to that for some other Arctic-breeding species experiencing sharp seasonal changes in environmental conditions (Lindholm et al. 1994). In birds breeding in northern environments, hatching synchrony is important due to a seasonal peak in food abundance (Visser et al. 1998; McKinnon et al. 2012). A decline in food supply throughout the season has been shown to be one of the main factors reducing growth and survival in late-hatched nestlings in other species (Daan et al. 1988; Brinkhof and Cavé 1997). Although this remains untested, we suggest that a seasonal decline in prey availability and vulnerability is likely the main factor explaining decreased survival of late-hatched falcon nestlings. Assuming adults initiate a clutch as soon as possible in the spring to avoid the cost of a delay (Rowe et al. 1994), they may face important trade-offs when selecting nest sites. Indeed, sites that offer the best sheltering capacity (e.g., caves and recesses) are present in the study area, but often remain packed with snow and are unavailable in early spring (A. Franke, personal observations). Falcons may then prefer to select sites well exposed to solar radiation and hence available in early spring, leaving nestlings more vulnerable to direct effect of rainfall during the brood-rearing period. This pattern is not unique to the study population and was observed in areas characterized by lower nest densities (A. Anctil and A. Franke, unpublished data), indicating that intra-specific competition is likely not the driving force explaining the use of unsheltered sites by most breeding birds.

Our results strongly suggest that the frequency of heavy rain has a much greater impact on nestling survival than the total amount of precipitation recorded during the rearing period. The latter parameter is, however, typically used in most ecological studies (e.g., Bradley et al. 1997; Lehikoinen et al. 2009). In our study system, direct observations showed that fatalities can occur in less than 2 h of heavy rain. The long-term precipitation data for our study site are consistent with the increase in extreme precipitation events noted in climate studies (e.g., Stone et al. 2000; Groisman et al. 2005) and it is predicted that the frequency of rainstorm events will continue to increase at a rapid pace, especially in the Arctic (Min et al. 2011). The negative effect of rainstorms on annual breeding productivity of Arctic-nesting falcons is therefore predicted to increase. However, a periodic boom in productivity due to a low frequency of heavy rain in a given year could allow population maintenance of a long-lived species like the peregrine falcon. Furthermore, although the direct effects of heavy rain explained an important proportion of the annual variation in nestling survival at our study site, other environmental factors, such as food availability (Potapov 1997; González et al. 2006), could strongly affect breeding success. Heavy rain could also interact with other factors and indirectly drive breeding productivity. Overall, the consequences of environmental change on population dynamics remain unknown and merit further study. Moreover, in order to better assess the impact of these changes at the regional and global scale, it would be important to understand the spatial variability in the vulnerability of falcons by conducting studies at other locations across their distribution range.

Birds and mammals have evolved powerful mechanisms to maintain their body temperature to avoid mortality (Hillenius and Ruben 2004). Hence, indirect effects of weather (i.e., through the food chain) are likely to play a larger role on their population dynamics than direct effects of weather (Berteaux and Stenseth 2006). However, young of many species are unable to maintain their body temperature (Evans 1984; MacArthur and Humphries 1999). Some species (e.g., cavity nesters, denning species and species that build elaborate nest structures) guard against inclement weather during their early stages of life and therefore might be expected to experience mainly the indirect effects of poor weather. Conversely, the young of other species that rely solely on parental brooding and chick huddling to maintain body temperature (Kirkley and Gessaman 1990), such as peregrine falcons, are much more likely to experience the direct effects of weather, especially during the critical period following birth. Variability in juvenile survival of long-lived vertebrates can play an important role in population dynamics (Gaillard et al. 1998). Hence, in species like the peregrine falcon, where rainfall can directly affect nestling survival, rapid changes in the precipitation regime could increase the vulnerability of the population. However, because long-lived species have delayed sexual maturity, the effects of climate change via reduced recruitment, if any, are unlikely to be noticed immediately due to a time-lag delay (Thompson and Ollason 2001).
